# 

**Published:** 1854-07-01

**Authors:** 


### To Correspondents.

Notwithstanding *two* additional sheets are given with this number, we are reluctantly compelled to postpone for the present the publication of several articles and reviews now in type. An elaborate analysis of a charming volume, entitled "Psychological Enquiries," will appear in our October number, along with critical notices of several French, American, and English journals and works. We had prepared for insertion a full report of the recent meeting of the "Association of Medical Officers of Hospitals for the Insane," held at Freemasons' Tavern, but cannot find space for its insertion. An account of the proceedings of this body will be published in the next number of the "Asylum Journal," and to this periodical we refer our readers. In justice to Dr. Jamieson, of Aberdeen, we are bound to state that the passage referring to the eccentricities of Dr. Johnson, quoted at the conclusion of the review that appeared in our last number of Dr. Mayo's Lectures, and Mr. Knaggs' work, was taken from that physician's "Lectures on the Medical Jurisprudence of Insanity." We were under an impression that Mr. Knaggs had quoted it, but we were in error.

